# Radiotherapy-induced diffuse myocardial fibrosis in early-stage breast cancer patients – multimodality imaging study with six-year follow-up

**DOI:** 10.1186/s13014-023-02319-z

**Published:** 2023-07-26

**Authors:** Mikko Moisander, Tanja Skyttä, Sari Kivistö, Heini Huhtala, Kjell Nikus, Vesa Virtanen, Pirkko-Liisa Kellokumpu-Lehtinen, Pekka Raatikainen, Suvi Tuohinen

**Affiliations:** 1 Faculty of Medicine and Health Technology, Tampere University, PO Box 100, Tampere, 33014 Finland; 2 Department of Oncology, Tampere University Hospital, Sädetie 6, PO Box 2000, Tampere, 33521 Finland; 3 Radiology, HUS Diagnostic Center University of Helsinki and Helsinki University Hospital, PO Box 100, Helsinki, 00029 Finland; 4 Faculty of Social Sciences, Tampere University, PO Box 100, Tampere, 33014 Finland; 5 Heart Hospital, Tampere University Hospital, PO Box 2000, Tampere, 33521 Finland; 6 Heart and Lung Center, Helsinki University Central Hospital and Helsinki University, PO Box 100, Helsinki, 00029 Finland

**Keywords:** Adjuvant Radiotherapy, Breast neoplasms, Cardiac Electrophysiology, Cardiotoxicity, Echocardiography, Endomyocardial Fibrosis, Multiparametric magnetic resonance imaging

## Abstract

**Background:**

Breast radiotherapy (RT) induces diffuse myocardial changes, which may increase the incidence of heart failure with preserved ejection fraction. This study aimed to evaluate the early signs of diffuse fibrosis after RT and their evolution during a six-year follow-up.

**Methods:**

Thirty patients with early-stage left-sided breast cancer were studied with echocardiography and electrocardiography (ECG) at baseline, after RT, and at three-year and six-year follow-up visits. Echocardiography analysis included an off-line analysis of integrated backscatter (IBS). ECG was analysed for fragmented QRS (fQRS). In addition, cardiac magnetic resonance (CMR) imaging was performed at the six-year control. The left ventricle 16-segment model was used in cardiac imaging, and respective local radiation doses were analysed.

**Results:**

Regional myocardial reflectivity in inferoseptal segments increased by 2.02 (4.53) dB (p = 0.026) and the percentage of leads with fQRS increased from 9.2 to 16.4% (p = 0.002) during the follow-up. In CMR imaging, abnormal extracellular volume (ECV) and T1 mapping values were found with anteroseptal and apical localization in a median of 3.5 (1.00–5.75) and 3 (1.25–4.00) segments, respectively. A higher left ventricle radiation dose was associated with an increased likelihood of having changes simultaneously in CMR and echocardiography (OR 1.26, 95% Cl. 1.00–1.59, p = 0.047).

**Conclusions:**

After radiotherapy, progressive changes in markers of diffuse myocardial fibrosis were observed in a multimodal manner in ECG and echocardiography. Changes in echocardiography and abnormal values in CMR were localized in the septal and apical regions, and multiple changes were associated with higher radiation doses.

**Supplementary Information:**

The online version contains supplementary material available at 10.1186/s13014-023-02319-z.

## Background

Radiotherapy (RT) remains an essential part of cancer therapy despite remarkable advances in medical cancer treatment. Refinements in RT treatment protocols have reduced adverse effects on healthy tissue, but some direct radiation and scattering unavoidably affects nearby tissue. Breast RT induces slowly evolving fibrotic changes in cardiac structures, causing excess late cardiac morbidity and mortality [1, 2].

The risk of heart failure with preserved ejection fraction (HFpEF) is increased 16-fold in breast cancer patients with prior RT compared to nonirradiated matched controls[3] Myocardial diffuse fibrosis and changes in diastology are found in patients with prior chest RT[1, 4] However, there is little knowledge about the evolution of diffuse fibrosis in patients treated with RT. In this study, the evolution and distribution of diffuse myocardial fibrosis were evaluated prospectively in a multimodal manner, including CMR imaging, echocardiography, and electrocardiography (ECG).

## Methods

### Patient selection

This single-centre prospective study included thirty eligible early-stage female left-sided breast cancer patients. The inclusion and exclusion criteria have been described previously in detail[5] In addition, a clinical contraindication for the CMR study was an exclusion criterion. The study was conducted from June 2011 to April 2019 at the Heart Hospital and Department of Oncology, Tampere University Hospital, Finland. The CMR studies were performed at the Heart Imaging Center, Helsinki University Hospital, at the end of the six-year follow-up period. The study complied with the Helsinki Declaration, and the local ethics committee approved the study protocol (R10160 and R11149). All participants signed an informed consent form before study enrolment.

### Radiotherapy

After surgery, all patients received adjuvant conformal 3D RT with modern RT techniques (3D-CRT). The treatment was delivered according to the institutional clinical guidelines. All patients underwent 3D computed tomographic treatment planning and were scanned under free breathing, as the voluntary deep-inspiration breath-hold technique (DIBH) was not implemented as clinical practice in our unit then. The radiation dose was either 50 Gy in 2 Gy fractions with an additional boost of 16 Gy in 2 Gy fractions to the tumor bed if clinically indicated or 42.56 Gy in 2.66 Gy fractions (hypofractionation). A mean heart dose constraint of 5 Gy was used in the treatment planning.

**Fig. 1:**
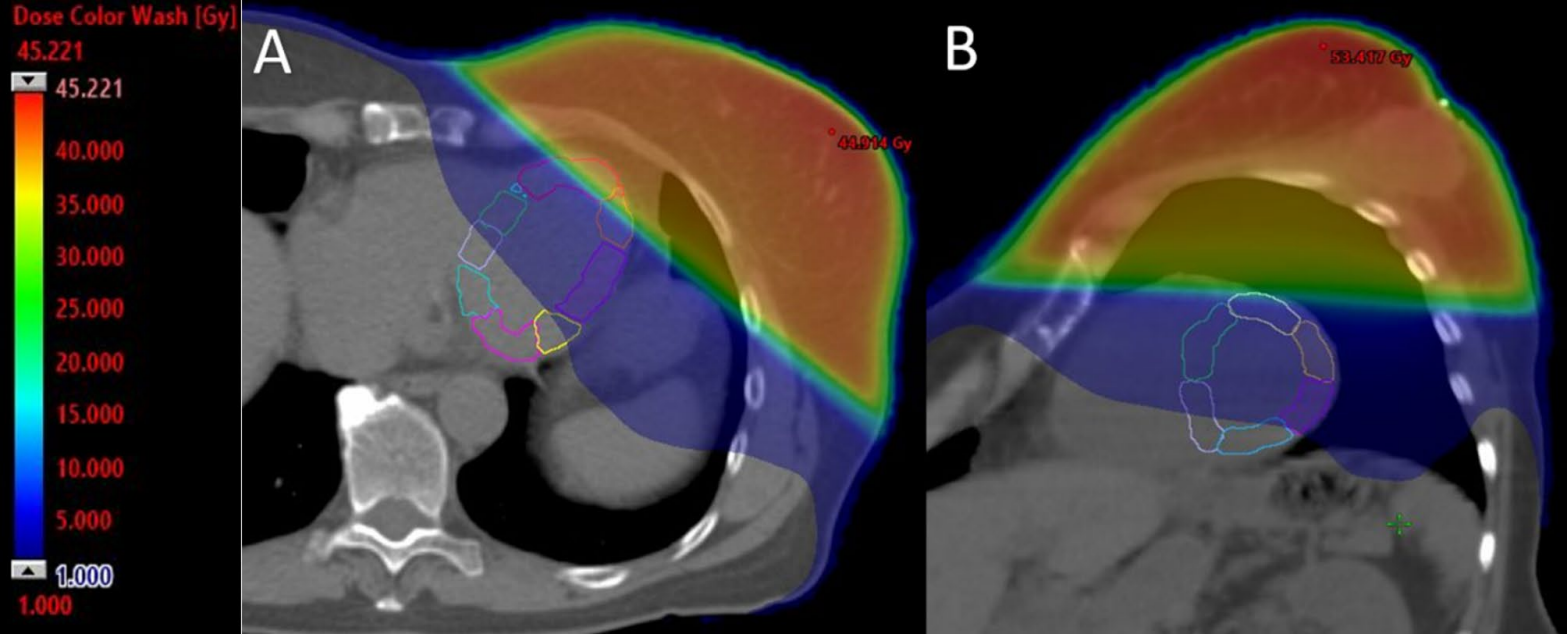
Radiotherapy fields in computed tomography treatment planning. Left ventricle segments are presented. Continuous color scale illustrates radiation doses received in the axial (A) and short-axis (B) views. Gy, gray

The full details of treatment planning and contouring of the primary heart structures have been described previously [6]. A dedicated clinical oncologist (MM) performed the left ventricle myocardial segment contouring with the 16-segment model, adapting the method described by Tang et al. as shown in Fig. 1 [7].

### Cardiac examinations

Cardiac examinations were performed 6 ± 8 days prior to RT, within three days of completion of the RT, and three (2.5–3.3 years) and six years (5.7–6.2 years) after RT. Echocardiography was performed using a 1–5 MHz matrix-array X5-1 transducer. Baseline to three-year control examinations were performed with a Philips iE33 ultrasound machine (Bothell, WA, USA). Due to the clinical update of equipment, the six-year control was performed using a Philips Epiq7 ultrasound machine (Bothell, WA, USA). The off-line analysis of integrated backscatter (IBS) was performed using Philips Qlab version 13 (Bothell, WA, USA). The IBS analysis is explained in Additional file 1: Figure S1. A change from baseline to the six-year control was used in the final analysis. An increase of ≥ 100% in reflectivity was assessed to be a significant change.

A 12-lead ECG was recorded at each study visit. A visible notch in any part of the QRS complex in several consecutive beats was defined as a fragmented QRS (fQRS) (Additional file 2: Figure S2). Each ECG was analysed by the same investigator (ST). All ECGs exhibited a normal sinus rhythm, and all the analysed QRS complexes had a duration less than 120 ms.

All study subjects underwent CMR imaging using a 3T Magnetom Skyra^fit^ system (Siemens, Erlangen, Germany). Cine-images were acquired using a balanced steady-state gradient echo (TrueFISP) sequence. All studies included standard long and short axis images. T1- and T2-mapping sequences were included in the standard protocol using a shortened Modified Look-Locker Inversion-recovery (ShMOLLI) sequence (Additional file 3: Figure S3). Pre- and 12 min post-gadolinium injection T1-mapping sequences were obtained to enable ECV measurements. All studies were analysed using Medis Suite Qmass, QStrain, T1 mapping, and T2/T2* mapping cardiac imaging programs (Medis Medical Imaging Systems, Leiden, The Netherlands).

### Statistical analysis

The data are presented as the means with standard deviations (SD) for variables with normal distributions, as medians with quartiles (Q_1_–Q_3_) for nonnormally distributed variables, or as numbers with percentages for categorical variables. The differences between groups were tested with the independent samples t-test or Mann–Whitney *U* test where appropriate. The differences in the measurements were tested with the paired samples t-test, Friedman’s test, Cochran’s Q test, or the Wilcoxon signed-rank test. Correlations were estimated using Spearman’s rank correlation coefficients. Multivariable linear regression analyses were performed to model the changes in fQRS, T1, ECV, and IBS values, adjusting the models with body mass index (BMI), hypertension, current smoking status, and mean left ventricle radiation dose. Binary logistic regression analysis was used to explain changes for groups formed based on the extent of abnormal imaging values. The analysis was performed with IBM SPSS Statistics software, version 27.0 for Windows (Armonk, NY, USA). All p-values are two-sided. All p-values less than 0.05 were considered significant.

## Results

### General characteristics

The baseline patient characteristics are shown in Table 1. All patients received RT as planned and completed the six-year follow-up uneventfully.

**Table 1:** Baseline characteristics

	Mean	SD
**Age (years)**	62.1	6.74
**BMI (kg/m** ^**2**^ **)**	26.58	4.23
**Systolic blood pressure (mmHg)** ^**a**^	151	19
**Diastolic blood pressure (mmHg)** ^**a**^	83	11
	**n**	**%**
**Smoking**		
Current	3	10
Previous	5	16.7
**Concurrent diagnosis**		
Hypertension^b^	11	36.7
Hypercholesterolemia	1	3.3
Hypothyreosis	5	16.7
No concurrent other diagnoses	16	53.3
**Baseline medication**		
β-blocker	3	10
ACE-inhibitors/ARBs	8	26.7
Diuretics	6	20
Statins	1	3.3
Aspirin	2	6.7
**Hormonal therapy**		
Use of aromatase inhibitor	5	16.7
Use of tamoxifen	1	3.3

BMI, body mass index; ACE-inhibitors, angiotensin-converting enzyme inhibitors; ARBs, angiotensin receptor blockers. ^a^ measured at the first visit, ^b^ defined as the medication-requiring state.

### Radiation doses

**Fig. 2:**
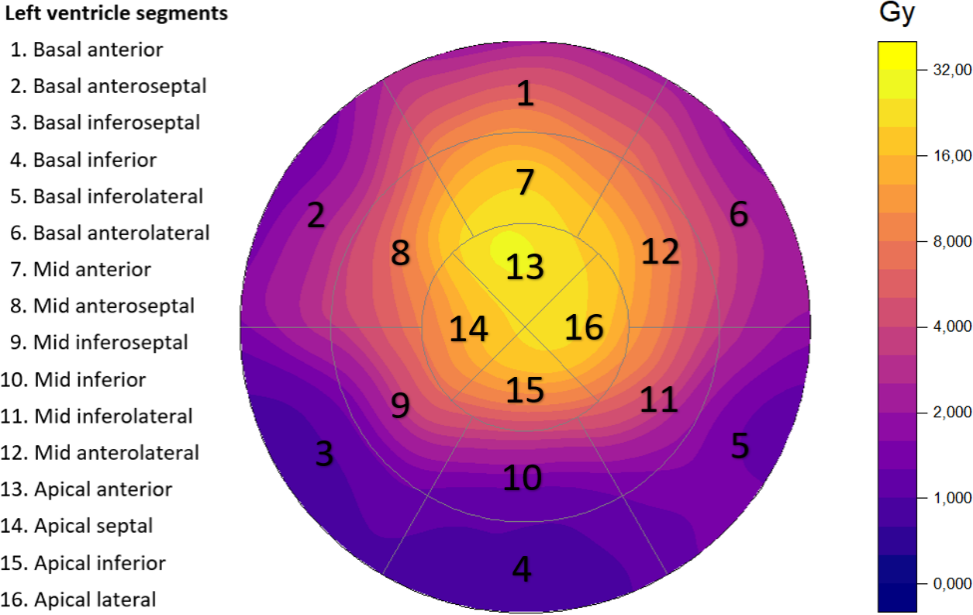
The mean segmental left ventricle radiation dose as a composite of data from all patients in a bulls-eye representation. The dose color map uses a log_2_ scale where yellow areas receive the highest dose and transition towards purple equals decreasing dose. Gy, gray

The mean heart radiation dose in the study population was 2.85 Gy (1.71–3.87 Gy). The mean heart dose was < 2 Gy in nine patients (30.0%), 2–4 Gy in 14 (46.7%), and > 4 Gy in seven (23.3%). The radiation doses varied remarkably between different left ventricle segments; please see Additional file 4: Table S1. The highest doses were in the apical and anterior segments, whereas the basal and inferior segments had the lowest doses. Figure 2 illustrates the distribution of radiation dose between the left ventricle segments.

### CMR

**Fig. 3:**
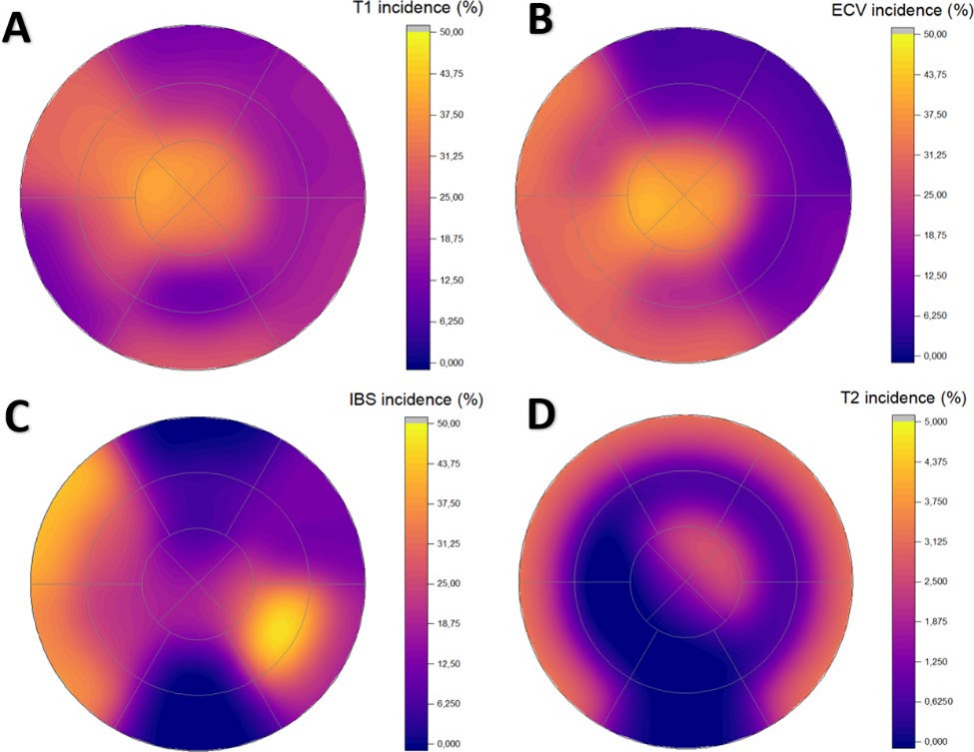
Incidence of abnormal segmental values of the left ventricle in a bulls-eye configuration. T1 (A), ECV (B), and IBS (C) values are presented according to the same scale (0 – 50%), whereas T2 is scaled from 0–5%. The same color scale is used for every variable where values increase while the color changes from purple to yellow. ECV, extracellular volume; IBS, integrated backscatter

At six years, CMR-assessed left ventricle end-diastolic and end-systolic volumes, ejection fraction, and mass were within normal range: 73.53 (15.75) mL/m^2^, 22.87 (7.31) mL/m^2^, 69.13 (7.04) %, and 48.53 (8.86) g/m^2^, respectively. In Table 2, regional values are presented. No significant late gadolinium enhancement (LGE) was observed in any of the patients. The incidence of abnormal segmental findings is presented in Fig. 3. For segmental values, please see Additional file 5: Table S2.

**Table 2:** Global and regional T1, ECV, T2, and IBS values

			Global	Basal	Mid	Apical	p
T1	(ms)	median (Q_1_-Q_3_)	1235 (1199–1273)	1220 (1193–1262)	1219 (1179–1268)	1252 (1168–1319)	0.393 ^1^
		*% abnormal	90.00	53.33	50.00	66.67	0.529^2^
ECV	(%)	median (Q_1_-Q_3_)	28.05 (26.11–30.23)	27.40 (25.23–28.78)	26.69 (24.87–29.29)	28.98 (26.65–32.51)	**0.008** ^1^
		*% abnormal	80.00	46.67	43.33	60.00	**0.038** ^2^
T2	(ms)	median (Q_1_-Q_3_)	35.54 (34.61–38.23)	35.82 (34.29–37.39)	34.88 (34.03–36.97)	36.85 (34.40–41.03)	**0.010** ^1^
		*% abnormal	6.90	3.45	0.00	3.45	0.368^2^
IBS	(dB)	mean (SD)	1.04 (2.80)	1.99 (3.93)	1.06 (3.73)	-0.79 (4.16)	0.080^1^
		*% abnormal	76.67	46.15	61.54	32.14	0.368^2^

One patient’s T2 values were missing, and two to four patients’ global or regional IBS values were missing and could not be evaluated. ECV, extracellular volume; IBS, integrated backscatter change from baseline to 6-year follow-up; *% abnormal, percentage of patients with abnormally high segmental values in a respective region; dB, decibel; p, p-value from ^1^Friedmans test or ^2^Cochran’s Q test for the difference between the basal, mid, and apical regions.

T1 mapping. The global median T1 was 1235 ms (1199–1273 ms). The incidence of abnormal T1 values (> 1300 ms) varied between segments, being most frequent in the apical and anteroseptal segments. Twenty-seven patients (90%) had at least one segment with abnormal T1 values (range 1–12). The median for number of abnormal segments was 3.00 (1.25–4.00), corresponding to 18.75% (7.81–25.00%) of the segments. In the multivariable analysis, hypertension predicted higher T1 values in apical segments (β = 0.461, p = 0.017).

T2 mapping. The global median T2 was 35.54 ms (34.61–38.23 ms). Only two patients (6.67%) had abnormal values (> 60 ms) in any of the segments. One patient had abnormal values in two apical segments, the other in five basal segments.

ECV mapping. The global ECV was 28.0% (26–30.2%). The incidence of abnormal ECV values (> 30%) varied between different segments, being most frequent in the apical and septal segments. In total, twenty-four patients (80%) had abnormal ECV values in at least one of the segments (range 1–13). The median number of abnormal segments was 3.50 (1.00–5.75), corresponding to 21.88% (6.25–35.94%) of the segments. In the multivariable analysis, hypertension predicted higher ECV values in apical segments (β = 0.416, p = 0.030).

### Integrated backscatter

The change from baseline to six-year follow-up visit was measured with IBS analysis. The global IBS change was 1.04 (2.80) dB (p = 0.060). The most significant change was seen in inferoseptal segments, from 9.29 (3.35) dB at baseline to 11.76 (4.30) dB at six years (p = 0.026). Values were regarded as abnormal if they increased ≥ 100%. Twenty-three patients (77%) had at least one segment with abnormal IBS values (range 1–6). The median number of abnormal segments was 1.00 (1.00–3.00), corresponding to 6.25% (6.25–18.75%) of the segments. In the multivariable analysis, BMI (β = 0.589, p = 0.009) and hypertension (β = -0.382, p = 0.042) predicted IBS change when the whole left ventricle was assessed.

### Fragmented QRS

Eighteen patients (60%) had fQRS in at least one lead (range 1–4 leads) at baseline. A spontaneous correction of some or all fragmentation changes was observed in twelve patients during the six-year follow-up.

**Fig. 4:**
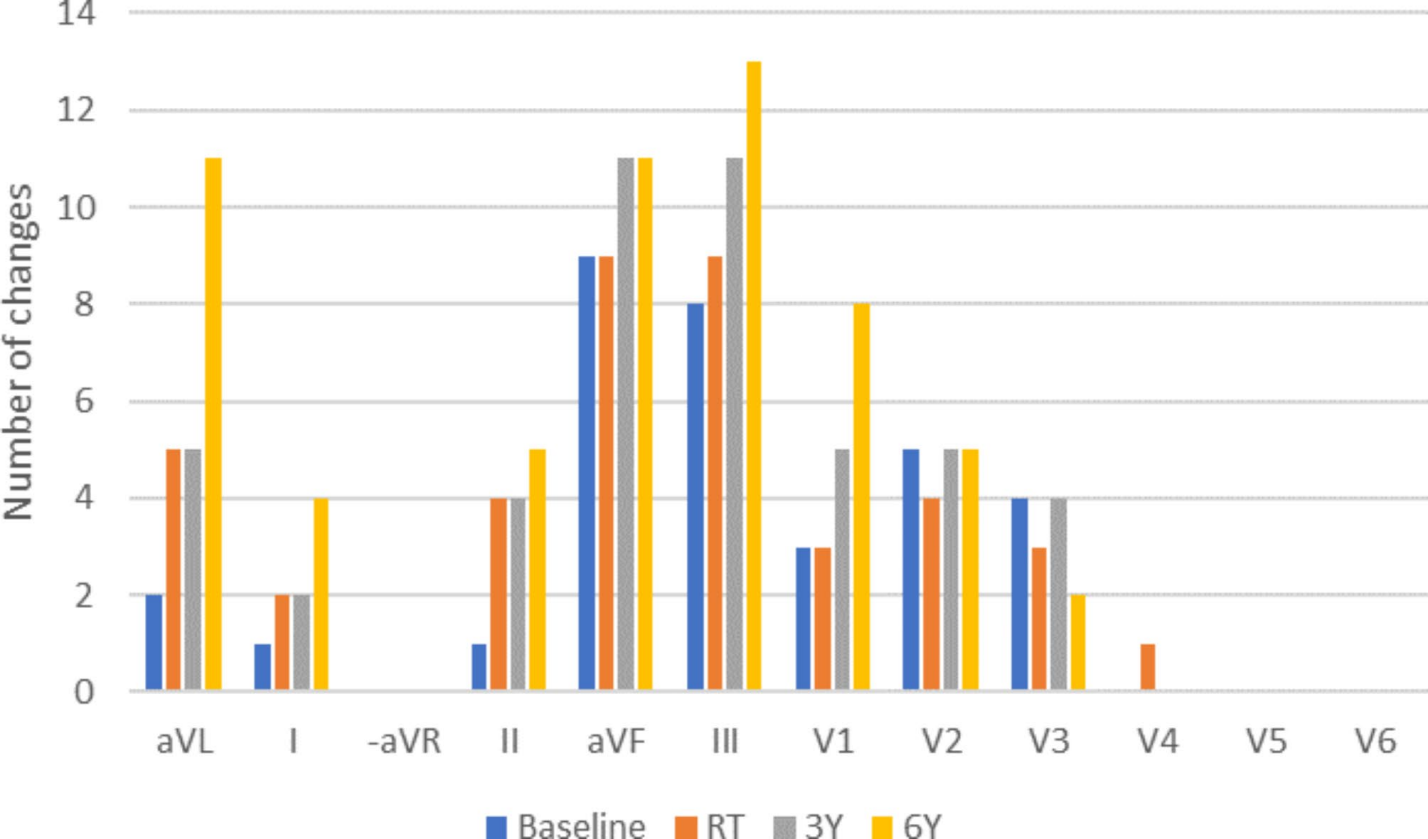
Fragmented QRS during the six-year follow-up. The leads are arranged according to the Cabrera lead system in order to present the changes in anatomical order

At six years, twenty-four patients (80%) had fQRS in one to five leads, including eight patients (26.7%) without fQRS at baseline. Novel changes (range 1–5 leads) were seen in twenty (66.7%) patients’ ECGs and the total percentage of leads with fQRS increased from 9.2 to 16.4% (p = 0.002) during the follow-up. Fourteen patients (46.7%) had new fragmentation at least in two different leads. For the distribution of changes, please see Fig. 4.

Patients were analysed based on whether they had more than one lead with a new fQRS during the six-year follow-up. The group with multiple new fragmentation changes (n = 14) had a statistically lower left ventricle radiation dose (3.77 vs. 8.04 Gy, p = 0.015) and were less frequently hypertensive (29% vs. 44%, p = 0.389). A higher left ventricle dose was associated with fewer new fQRS in the multivariable analysis (β = -0.569, p = 0.013).

### Regional analysis combining multiple changes

Additional analyses were performed considering the extent of abnormal values in T1, ECV, and IBS in the different segments. There was no correlation between individual segmental or regional changes and RT doses. However, five patients (16.7%) had simultaneous abnormal values in T1, ECV, and IBS in the same segments. The patients with abnormal values in all three analyses had a higher left ventricle dose (8.04 Gy vs. 4.74 Gy, p = 0.108) than the rest, although this did not reach statistical significance. In the binary logistic regression analysis, increasing left ventricle radiation dose was associated with an increase in the likelihood of having changes in all three measurements (OR 1.26, 95% Cl. 1.00–1.59, p = 0.047).

## Discussion

This multimodality imaging study showed that changes indicating diffuse myocardial fibrosis were progressive (IBS and ECG) during a six-year follow-up period, and they were localized in the RT-prone septal and apical segments in the echocardiography and CMR analysis. An association with radiation dose was seen when multiple parameters were combined.

### Radiotherapy-induced myocardial changes

A key mechanism of RT-induced cardiotoxicity is the development of diffuse myocardial fibrosis[1] Histological studies have verified the development of fibrosis in later phases after chest RT, with a more severe impact after a higher radiation dose [1, 8].

Diffuse fibrosis is commonly present in the early phases of several heart disorders, and the extent of fibrosis has been associated with the severity of clinical symptoms[9] In fact, Saiki et al. have shown that 8% of breast cancer patients treated with RT developed heart failure within six years of the treatment[3] Most commonly, patients presented with HFpEF and a higher RT dose was associated with elevated end-diastolic pressure and left ventricular stiffness[3, 4] Therefore, the development of diffuse myocardial fibrosis after breast RT has a high clinical importance, and its early recognition is prudent for prognostic and therapeutic reasons. However, there is a lack of studies assessing these modalities in patients treated with RT without chemotherapy.

### Diffuse myocardial fibrosis in CMR

The role of CMR in oncology has been established, and growing evidence endorses the use of CMR in evaluating cancer patients receiving systemic cancer therapy[10] Currently, CMR is regarded as the radiological gold standard in determining diffuse myocardial fibrosis, characterized by an increase in native T1 and ECV values, which correlate with histological fibrosis findings [10, 11].

Studies of patient groups receiving chemotherapy and RT include lymphoma and breast, lung, and oesophagus cancer patients[12–17] It seems that the first year after the treatment is characterized by an increase in T1 and T2 values in CMR without a clear RT dose relationship, possibly presenting only oedematous tissue change in the early phase[15, 17] In later phases after RT, an increase in ECV has been observed with a linear dose association with segmental cardiac radiation doses in a study by de Groot et al.[12] Furthermore, Bergom et al. showed a correlation between a higher cardiac RT dose and myocardial mass in breast cancer patients[16] Therefore, it seems that while tissue oedema changes dominate the first year, an increase in myocardial mass and ECV becomes evident in the later phase, indicating the evolution of diffuse fibrotic changes. However, all these studies included patients with combination therapies.

In our study, 90% and 80% of the patients (n = 27 and n = 24) presented abnormal segmental values in T1 and ECV mapping, respectively. The proportion of abnormal myocardial segments was 18.75% in T1 mapping and 21.88% in ECV mapping, a significant proportion concentrated in the apical and anteroseptal areas. As RT is known to induce diffuse myocardial fibrosis, our finding of changes indicating diffuse myocardial fibrosis in the areas of higher radiation dose is noteworthy. However, a dose relationship with a segmental radiation dose was not evident comparing single parameters, as has been the case in other studies [14, 15].

While 36.7% of our patients had hypertension at baseline and an increase in the T1 and ECV values have been observed in hypertensive patients, this acts as a possible confounding factor. However, T1 and ECV elevation has been remarkable only among patients with co-occurring left ventricular hypertrophy, which was not present in our study population[18] Besides apical segments, we found no association between hypertension and T1 or ECV. There is no evidence that hypertension would cause isolated CMR changes; therefore, RT is the most plausible explanation for the recorded abnormal values [18].

### Integrated backscatter

Integrated backscatter measures myocardial reflectivity. The first studies showed increased values with histologically verified replacement scars[19] Later studies indicate that myocardial reflectivity increases for various reasons, including hypertrophic cardiomyopathy and ischemia[20, 21] In our previous study, we showed that myocardial reflectivity increases after RT in anterior RT-prone areas in contrast to posterior parts [22].

In this study, all left ventricle segments were studied. A doubling of reflectivity was observed in 77% of our patients in at least one segment. Overall, 6.3% of the segments displayed a doubling of myocardial reflectivity, indicating the evolution of myocardial changes. Rising baseline BMI predicted a more significant change in left ventricle IBS values in our study. Obesity has been previously linked to higher IBS values, and it may predispose the heart to the cardiotoxicity of RT [23, 24].

### Fragmented QRS

The presence of fQRS has been associated with abnormal myocardial activation and has been investigated in multiple cardiac diseases[25] It has been shown that fQRS holds poor prognostic features[26] An association between fQRS and diffuse fibrosis has been established previously, showing an association between fQRS and CMR findings[27] Furthermore, Konno et al. found that the number of leads with fQRS was associated with the extent of CMR findings in hypertrophic cardiomyopathy patients, indicating that fQRS can be used both qualitatively and quantitatively to detect myocardial fibrosis [28].

Knowledge about fQRS after RT is scarce. In one study, Adar et al. [29]. followed 52 breast cancer patients treated with RT. In total, 37% of the patients developed a new fQRS during a one-year follow-up. They also observed an association between RT dose and the development of fQRS. However, 86% of their patients also received chemotherapy. In our study, a total of 66.7% of the patients accumulated new fragmentation changes, and half of the patients (47%) had fQRS in multiple leads. The prevalence of leads with fQRS increased from 9.2 to 16.4%. To our knowledge, this is the first study to present a progressive accumulation of fQRS after solely RT-treated patients.

We found an unexpected inverse association between new fQRS and radiation dose. Patients with multiple new fQRS also had hypertension less frequently, although this was not statistically significant. While it is counter-intuitive that a higher RT dose would cause fewer changes in the ECG, it can be speculated whether the hypertension medication protected patients from RT-induced cardiotoxicity, as we know that these medications hold cardioprotective qualities[30] In addition, this could explain why hypertension predicted a decrease in IBS. Additionally, the cancellation of ECG changes in anatomically opposite myocardial segments with advancing myocardial changes could explain the seemingly counter-intuitive results.

### Association of multiple changes with left ventricle radiation dose

We found an association with left ventricle RT dose in the situations where abnormal values were observed in the same segment with CMR (T1 and ECV) and echocardiography (IBS). This was most often seen apically and anteroseptally, where the high RT doses were delivered. However, no direct segmental level correlation was found. This might be due to various reasons, including imprecision between various imaging modalities considering the segmental data level. However, an association with the radiation dose was evident when the impact was high enough to simultaneously induce changes in the same segment in both CMR and echocardiography. It may be that milder impact was diluted by imaging inaccuracies, while only a more powerful effect with multiple changes produced an association with radiation heart doses.

### Clinical implications

Our study endorses the results of previous studies indicating the evolution of diffuse myocardial fibrosis after RT [12, 13, 15] In addition, our study is the first prospective follow-up study concentrating on findings of diffuse fibrosis in patients treated with postoperative RT without chemotherapy, showing its progressive nature. The early recognition of RT-induced myocardial fibrosis might open a window for effective early treatment, including the diligent management of other contributing factors, such as hypertension, as well as new specific treatment choices for HFpEF [31].

### Limitations

There are several limitations to be acknowledged. *First*, the study population was small and not powered enough to show a statistically meaningful correlation. *Second*, the results did not translate to clinically significant adverse events, most likely due to too short follow-up time. *Third*, there is evidence that cancer itself can cause changes in the myocardium shown by the CMR[32] This could not be excluded since there was no baseline CMR imaging. *Fourth*, there were technical issues with the imaging. In contrast to CMR and echocardiography imaging, CT images were not ECG-gated or acquired with a breath-hold. This might have contributed to the missing segmental associations. In addition, we acknowledge that IBS is an angle-sensitive measurement also affected by depth. However, to overcome difficulties caused by angle and depth, a relative change in tissue reflectivity during the follow-up period was used instead.

## Conclusion

During a six-year follow-up, multimodality imaging identified an accumulation of the markers indicating diffuse fibrosis in RT-prone regions of the left ventricle. An association with RT dose was found when multiple modalities were combined.

## Electronic supplementary material

Below is the link to the electronic supplementary material.

Additional file 1: Figure S1. The integrated backscatter off-line analysis.

Additional file 2: Figure S2. Fragmented QRS analysis example.

Additional file 3: Figure S3. T1, T2 and extracellular volume (ECV) mapping in the CMR imaging.

Additional file 4: Table S1. Mean and maximal radiation doses of left ventricle segments.

Additional file 5: Table S2. Left ventricle segmental T1, T2, ECV, and IBS values.

## Data Availability

The datasets used and analyzed during the current study are available from the corresponding author on reasonable request.

## Abbreviations

BMI: Body mass index
CMR: Cardiac magnetic resonance
ECG: Electrocardiography
ECV: Extracellular volume
fQRS: Fragmented QRS
HFpEF: Heart failure with preserved ejection fraction
IBS: Integrated backscatter
RT: Radiotherapy
SD: standard deviation
ShMOLLI: Shortened Modified Look-Locker Inversion-recovery
