# Supplementary material for: Radiotherapy-induced diffuse myocardial fibrosis in early-stage breast cancer patients – multimodality imaging study with six-year follow-up
Source: Radiat Oncol. 2023 Jul 26;18:124. doi: 10.1186/s13014-023-02319-z (PMC10373367; doi:10.1186/s13014-023-02319-z)
Supplement: Supplementary file 1 — Additional file 1: Figure S1. The integrated backscatter off-line analysis. [file 13014_2023_2319_MOESM1_ESM.pdf]

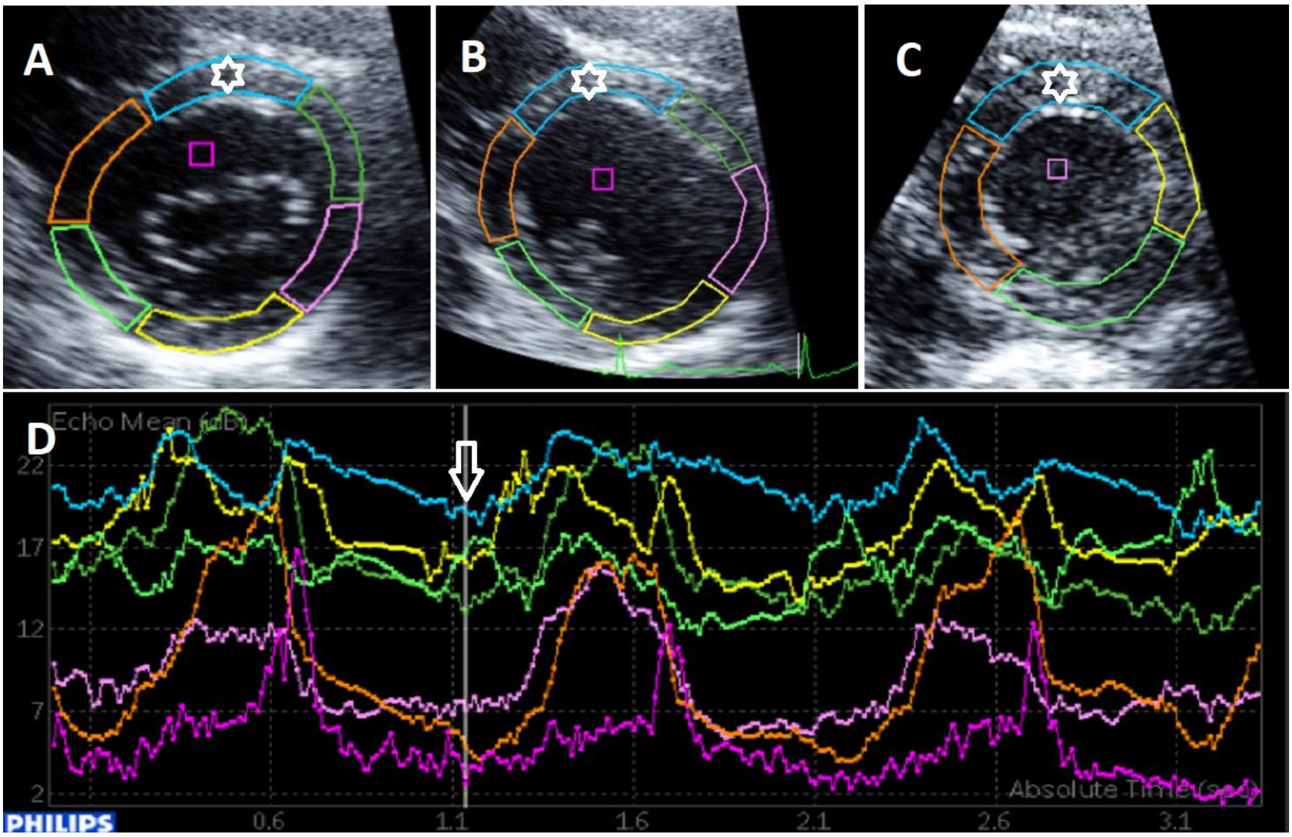

Figure S1. The integrated backscatter off-line analysis. The short axis basal (A), mid (B), and apical (C) images were opened in the Qlab software 'Region of interest' analysis program, and regions corresponding CMR segmentations are manually drawn on the left ventricular myocardium carefully avoiding bright endo- and epicardial speckles. The anterior segments are marked with a white star in images A-C. Segmentations counterclockwise from the anterior segment in images A and B are anteroseptal, inferoseptal, inferior, inferolateral, and anterolateral. In image C, the respective segments are septal, inferior, and lateral. Each image also contains a blood pool reference box in the middle (pink box). The lower part of the image illustrates the results of the myocardium reflectivity from the basal short-axis image. The measurement is performed at the end-diastole defined by the beginning of the QRS-complex and marked by the white arrow in image D. The final value for each segment is calculated by subtracting the segmental value and the reference blood pool value.
