# Supplementary material for: Radiotherapy-induced diffuse myocardial fibrosis in early-stage breast cancer patients – multimodality imaging study with six-year follow-up
Source: Radiat Oncol. 2023 Jul 26;18:124. doi: 10.1186/s13014-023-02319-z (PMC10373367; doi:10.1186/s13014-023-02319-z)
Supplement: Supplementary file 2 — Additional file 2: Figure S2. Fragmented QRS analysis example. [file 13014_2023_2319_MOESM2_ESM.pdf]

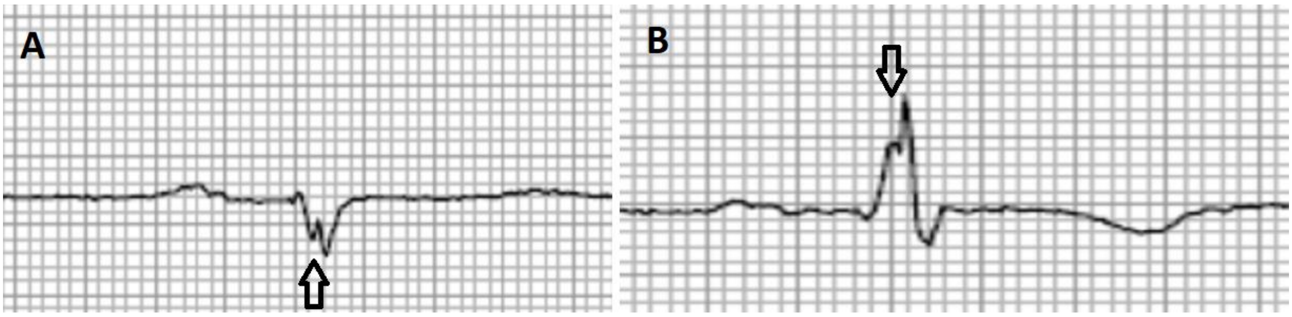

Figure S2. Fragmented QRS analysis example. A clear notch in any part of the QRS complex was registered, marked with an open arrow in the descending (A) and ascending (B) parts of the QRS complex.
