# Supplementary material for: Radiotherapy-induced diffuse myocardial fibrosis in early-stage breast cancer patients – multimodality imaging study with six-year follow-up
Source: Radiat Oncol. 2023 Jul 26;18:124. doi: 10.1186/s13014-023-02319-z (PMC10373367; doi:10.1186/s13014-023-02319-z)
Supplement: Supplementary file 3 — Additional file 3: Figure S3. T1, T2 and extracellular volume (ECV) mapping in the CMR imaging. [file 13014_2023_2319_MOESM3_ESM.pdf]

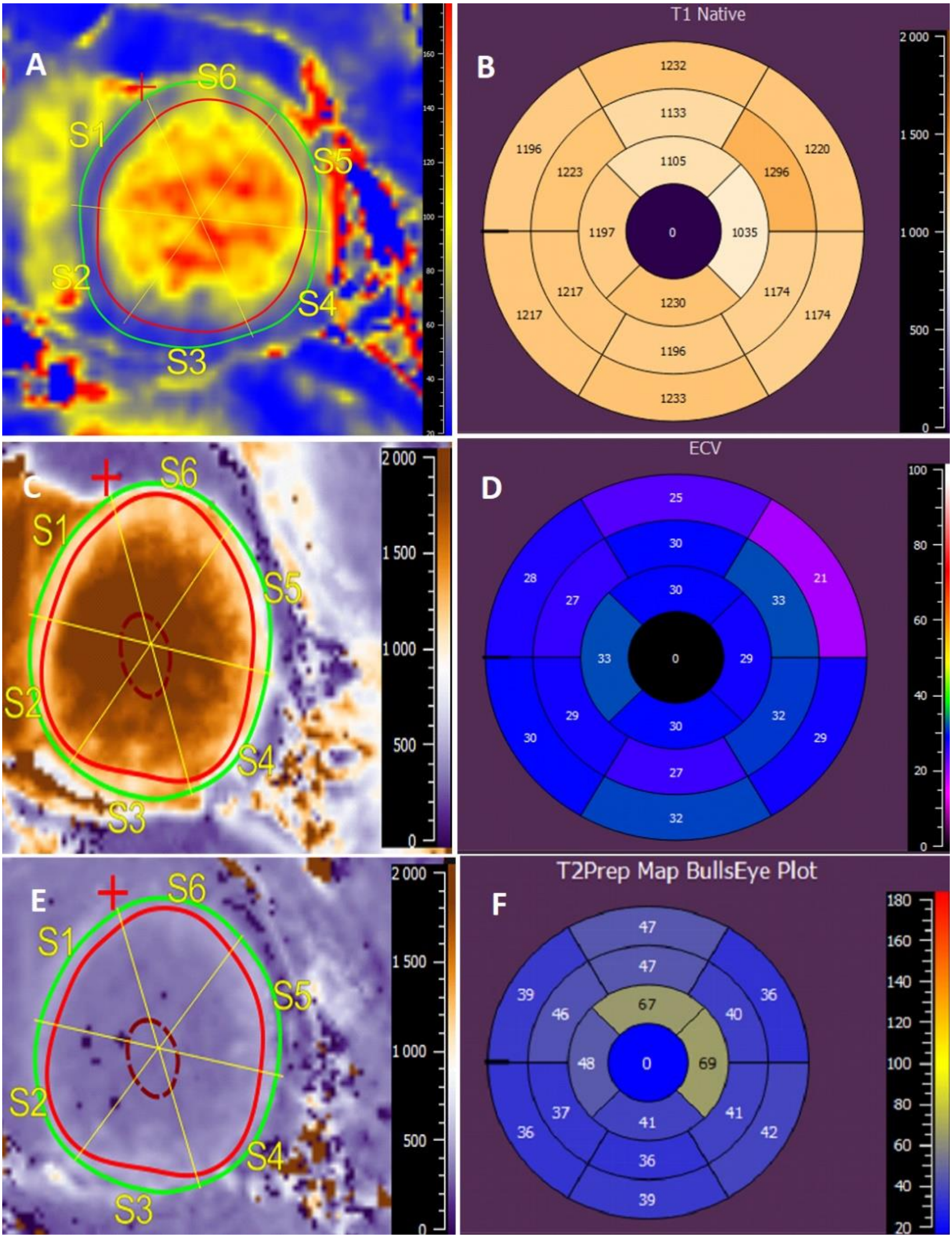

Figure S3. T1, T2 and extracellular volume (ECV) mapping in the CMR imaging. Cross-sectional images from basal, mid and apical levels of the left ventricle were derived with and without Gadolinium contrast injection (A, C and E). An off-line analysis with dedicated software was performed and results in bulls-eye configuration for T1 (B), ECV (D) and T2 (F) were formed for each patient.
