# Supplementary material for: Radiotherapy-induced diffuse myocardial fibrosis in early-stage breast cancer patients – multimodality imaging study with six-year follow-up
Source: Radiat Oncol. 2023 Jul 26;18:124. doi: 10.1186/s13014-023-02319-z (PMC10373367; doi:10.1186/s13014-023-02319-z)
Supplement: Supplementary file 4 — Additional file 4: Table S1. Mean and maximal radiation doses of left ventricle segments. [file 13014_2023_2319_MOESM4_ESM.pdf]

Table S1. Mean and maximal radiation doses of left ventricle segments.

Left ventricle segment	Radiation dose (Gy)	
	Mean	Max
Basal anterior	2.74 (2.33–3.57)	5.07 (3.88–7.36)
Basal anteroseptal	1.81(1.38–2.18)	3.27 (2.58–4.04)
Basal inferoseptal	0.97 (0.76–1.25)	1.63 (1.19–1.95)
Basal inferior	0.81 (0.62–1.06)	1.17 (0.87–1.47)
Basal inferolateral	1.31 (1.07–1.62)	2.08 (1.84–2.90)
Basal anterolateral	2.17 (1.95–2.84)	3.85 (3.32–5.03)
Mid anterior	9.18 (4.97–15.70)	38.57 (24.39–44.56)
Mid anteroseptal	3.22 (2.19–5.08)	21.45 (5.40–37.84)
Mid inferoseptal	1.49 (1.01–2.03)	2.68 (1.70–3.16)
Mid inferior	1.25 (0.92–1.80)	1.96 (1.42–2.74)
Mid inferolateral	2.05 (1.55–2.55)	4.02 (2.79–6.15)
Mid anterolateral	4.23 (3.08–7.72)	23.55 (7.75–36.91)
Apical anterior	29.51 (12.59–38.88)	45.43 (38.92–47.30)
Apical septal	9.33 (4.02–18.52)	37.23 (18.39–44.61)
Apical inferior	6.51 (2.72–10.63)	13.30 (9.56–25.47)
Apical lateral	13.73 (4.43–26.02)	41.01 (26.15–45.64)
p	**<0.001**	**<0.001**

Values are median (Q_1-Q_3). Gy, gray; p, p-value from Friedmans test testing the difference of radiation dose between different left ventricle segments. Statistical significance is shown in bold ($p < 0.05$).
