# Supplementary material for: Radiotherapy-induced diffuse myocardial fibrosis in early-stage breast cancer patients – multimodality imaging study with six-year follow-up
Source: Radiat Oncol. 2023 Jul 26;18:124. doi: 10.1186/s13014-023-02319-z (PMC10373367; doi:10.1186/s13014-023-02319-z)
Supplement: Supplementary file 5 — Additional file 5: Table S2. Left ventricle segmental T1, T2, ECV, and IBS values. [file 13014_2023_2319_MOESM5_ESM.pdf]

Table S2. Left ventricle segmental T1, T2, ECV, and IBS values.

	T1<br>ms	T2<br>ms	ECV<br>%	IBS<br>dB
Basal anterior	1194 (1162–1230)	35.30 (33.40–37.20)	25.1 (23.3–27.1)	1.05 (5.15)
Basal anteroseptal	1228 (1195–1312)	35.30 (34.30–37.70)	28.2 (26.6–30.5)	1.54 (6.60)
Basal inferoseptal	1222 (1207–1275)	36.40 (34.55–40.10)	28.5 (26.3–31.2)	3.64 (6.24)
Basal inferior	1255 (1219–1323)	35.30 (33.55–38.20)	27.6 (26.3–31.9)	1.10 (4.60)
Basal inferolateral	1210 (1157–1283)	34.90 (34.05–36.60)	27.5 (25.8–28.9)	1.90 (4.05)
Basal anterolateral	1187 (1156–1254)	34.50 (33.35–36.55)	25.5 (23.1–26.8)	2.55 (5.71)
Mid anterior	1176 (1117–1236)	35.20 (34.15–38.10)	25.5 (22.7–28.7)	0.15 (4.88)
Mid anteroseptal	1243 (1189–1351)	36.20 (33.30–39.00)	27.3 (25.9–29.9)	0.92 (5.28)
Mid inferoseptal	1231 (1184–1311)	34.60 (33.55–37.20)	27.5 (24.0–31.4)	2.53 (6.14)
Mid inferior	1227 (1179–1271)	34.20 (32.70–35.80)	26.9 (24.8–29.1)	-1.62 (6.43)
Mid inferolateral	1193 (1147–1273)	34.00 (33.35–36.30)	27.0 (24.3–28.9)	2.49 (3.73)
Mid anterolateral	1192 (1152–1268)	35.30 (33.90–37.55)	25.6 (23.5–28.4)	2.21 (5.52)
Apical anterior	1236 (1128–1329)	37.90 (36.00–40.80)	27.9 (25.3–33.7)	-1.56 (4.94)
Apical septal	1248 (1206–1327)	36.80 (34.25–43.10)	29.4 (27.4–34.1)	0.03 (7.52)
Apical inferior	1245 (1173–1337)	34.20 (33.00–37.35)	28.3 (25.9–33.6)	-1.26 (5.89)
Apical lateral	1221 (1127–1312)	37.20 (34.05–43.00)	28.7 (25.8–32.2)	-1.63 (4.67)
p-value	**<0.001**	**<0.001**	**<0.001**	**<0.001**

Values are median (Q_1-Q_3) for T1, T2 and ECV, and mean (SD) for IBS. Three to twelve patients' IBS values per segment were missing and could not be evaluated. One patient's segmental T2 values were missing and could not be evaluated. ECV, extracellular volume; IBS, integrated backscatter, change from baseline to six-year follow-up visit; dB, decibel; p, p-value from Friedmans test testing the difference between different left ventricle segments. Statistical significance is shown in bold ($p < 0.05$).
